# Supplementary material for: Elevated Expression of CCN3 in Articular Cartilage Induces Osteoarthritis in Hip Joints Irrespective of Age and Weight Bearing
Source: Int J Mol Sci. 2022 Dec 4;23(23):15311. doi: 10.3390/ijms232315311 (PMC9738275; doi:10.3390/ijms232315311)
Supplement: Supplementary file 1 [file ijms-23-15311-s001.zip › ijms-2069853-supplementary.pdf]

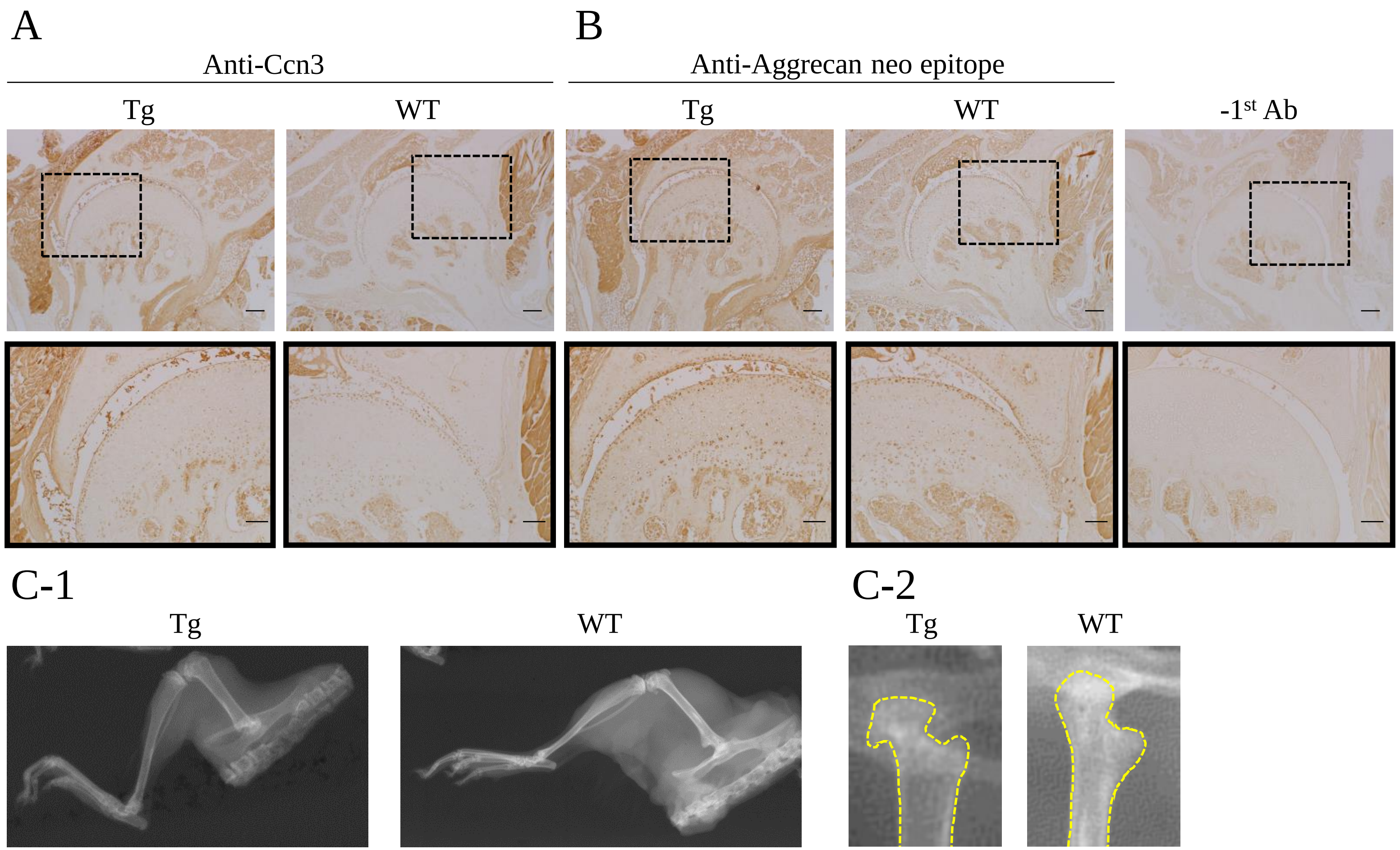

Supplemental figure S1

Immunohistochemical staining of **(A)** Ccn3 and **(B)** Aggrecan neo epitope in femoral head of 3 month-old Ccn3 Tg and WT mice. The black framed photos are magnification of the dashed box in the photos above. **(C)** X-ray images of (C-1) lower extremity) and (C-2) femoral head of the 2 month-old Ccn3 tg and WT. Bar: 100μm
